# A Comparison of Different Biomechanical Systems for the Orthodontic Treatment of Palatally Impacted Canines

**DOI:** 10.3390/bioengineering12030267

**Published:** 2025-03-09

**Authors:** Ioannis P. Zogakis, Chrysanthi Anagnostou, Ioulia Ioannidou, Stella Chaushu, Moschos A. Papadopoulos

**Affiliations:** 1 Department of Orthodontics, School of Dentistry, Aristotle University of Thessaloniki, 54124 Thessaloniki, Greece; 2 Department of Hygiene, Social-Preventive Medicine and Medical Statistics, School of Medicine, Aristotle University of Thessaloniki, 54124 Thessaloniki, Greece; 3 Department of Orthodontics, Hebrew University of Jerusalem, Hadassah Medical Center, Jerusalem 91120, Israel

**Keywords:** impacted canine, displaced canine, ballista spring, cantilever, attachment

## Abstract

Canine impaction constitutes a clinical entity that, if untreated, can compromise dentition. Thus, various treatment approaches and different biomechanical systems have been proposed over the years for its management. Clinical records of patients who consecutively visited the Postgraduate Clinic of the Department of Orthodontics of the Aristotle University of Thessaloniki, Greece, were retrieved and analyzed retrospectively with the aim to compare two different biomechanical systems for the orthodontic/surgical treatment of palatally displaced canines. A total of 29 patients with 36 palatally impacted canines were included in the current investigation. The patients had a median age of 16 years (IQR: 15–20); 69% of them were females (n = 20) and 31% were male (n = 9). No statistically significant differences regarding treatment outcomes were detected between different types of active unit (*p* > 0.99), or the types of bonded attachments (*p* = 0.52). The use of ballista springs or cantilever configurations was not found to significantly affect the alignment duration (*p* = 0.56) as opposed to the type of attachment, where eyelets outmatch brackets (*p =* 0.009). The use of brackets over eyelets significantly prolongs the canine alignment duration.

## 1. Introduction

A tooth is considered to be impacted when more than three-quarters of its final root length have been developed, yet it is still not erupted or anticipated to erupt, based on clinical and radiographical findings [1]. Various etiological factors have been proposed as accountable for the impaction of a developing canine, including poor guidance by the root of the lateral incisor and hereditary factors [2]. Canine impaction is a clinical condition with a frequency that differs contingent on the population under investigation, with multiple studies documenting a range from 0.9 to 5%, with maxillary canine displacement being more frequent than mandibular displacement [2,3,4,5,6]. In terms of the position of impaction regarding the maxillary canines, numerous studies contend that palatal displacement is more frequent than labial displacement [3,4,7].

Considering that canine impaction constitutes a pathological condition that could lead to complications in relation to normal functional occlusion and dental aesthetics, its resolution is deemed imperative. Certain treatment options that aim for to the prevention of impaction or its resolution are available. The most common treatment strategy, should the impaction not be prevented by early interceptive treatment [8], consists of surgical uncovering, followed by orthodontic traction and alignment [9]. The purpose of surgical exposure is to provide access to the palatal surface of the impacted canine so that an attachment can be bonded, ideally by the treating orthodontist [9], and then be used to apply orthodontic extrusive forces by means of an active unit. The ultimate goal of orthodontic/surgical treatment is for the impacted canine to successfully reach the dental arch and to be fully aligned.

Various biomechanical systems, including cantilever configurations, ballista springs, elastics, and piggyback archwires as active units, as well as different bonded attachments, such as brackets, eyelets, and buttons, have been widely used by orthodontists, aiming at the resolution of impactions [10]. Ballista springs, first described by Jacoby in 1978, are generally fabricated by a round stainless-steel wire, with one end inserted inside the first molar tube, and the other end ligated to the impacted canine after being bent 90 degrees towards the lower arch [11]. Further, cantilever configurations constitute simple orthodontic appliances usually consisting of a wire, where one end is attached to an anchor unit in the posterior segment, and the other end is ligated to the impacted canine.

Over the years, several studies investigating factors that influence the outcome and duration of the orthodontic/surgical treatment of ectopic canines, mainly focusing on patient-related factors and the choice of surgical exposure technique by the clinicians, have been published [12,13,14,15,16,17,18,19,20,21,22,23]. However, to the authors’ best knowledge, no clinical study comparing these biomechanical systems, and more specifically cantilever configurations with ballista springs as active units and brackets with eyelets, and as bonded attachments, pertaining to the treatment outcome and duration, can be found in the literature. Consequently, the aim of the present study was to investigate the potential influence of these types of active units and bonded attachments on the different aspects of the orthodontic/surgical treatment of palatally impacted canines.

## 2. Materials and Methods

### 2.1. Study Design

This study was designed as a retrospective observational study, comparing different biomechanical systems employed for the orthodontic treatment of palatally displaced canines. More specifically, the purpose of the current investigation was to examine whether the use of a ballista spring or a cantilever configuration as an active unit, and the use of an eyelet or a bracket as a bonded attachment, during the orthodontic/surgical treatment of palatally impacted canines, had an impact on the treatment outcome and duration of canine alignment. Treatment was defined as “successful” if full alignment of the impacted canine in the dental arch was achieved, and as “failed” if the canine could not be fully aligned, presumably due to ankylosis to the bone surrounding it, or invasive cervical root resorption [24]. A slight rotation was considered acceptable if the canine reached full alignment [14]. The canine alignment duration was calculated in months and was defined as the time period from the date of the surgical exposure, during which active traction was applied, to the date that the canine reached the dental arch and was fully ligated into a nickel—titanium main archwire [22]. All of the patients’ clinical records were retrieved and evaluated by one experienced orthodontist (IPZ).

In this study, in terms of the active unit, a modification of the original ballista spring, as described by Jacoby in 1978 [11], consisting of a 0.016 inch stainless-steel auxiliary wire inserted in the slot of the adjacent brackets in a piggyback fashion, as proposed by Kornhauser et al., was implemented [25]. Further, the cantilever configurations used in the present study consisted of a 0.8 mm Blue Elgiloy^®^ wire (RMO, Denver, CO, USA) soldered to a 0.9 mm transpalatal arch (TPA) made of stainless steel, delivering a single-point contact extrusive force to the impacted canine.

In addition, the type of bonded attachment was investigated as a factor that could potentially affect the treatment outcome and/or the canine alignment duration. More specifically, the use of a standard edgewise bracket was compared to that of a simple eyelet, directly bonded to the palatal surface of the impacted canine during surgical exposure. The choice of one active unit and bonded attachment over the other was independent of the severity of impaction and depended solely on the clinician’s preferences.

### 2.2. Population

The subjects were patients who consecutively attended the Postgraduate Clinic of the Department of Orthodontics of the School of Dentistry of the Aristotle University of Thessaloniki, Thessaloniki, Greece, with at least one impacted canine, were referred for surgical exposure, and were treated by orthodontic extrusion and alignment, with complete clinical and treatment records. Patients for whom the treatment of choice was extraction of the displaced canine and those whose clinical records were incomplete were not included in this study. Patients exhibiting one or more condition that may influence the normal development of permanent dentition, including pathological conditions, hereditary diseases, or syndromes, were excluded from the current study. Further, patients with mandibular impacted canines or buccally displaced canines in the maxilla, as well as patients with canine impaction who underwent orthodontic/surgical treatment without using either a ballista spring or a cantilever as an auxiliary mechanism, were also excluded from the current investigation.

### 2.3. Ethics Approval

Prior to conducting this study, permission was obtained from the Bioethics Committee of the School of Dentistry of the Aristotle University of Thessaloniki, Greece (Nr. 25/21.11.2024).

### 2.4. Statistical Analysis

Data were analyzed using the open Jamovi software (Version 2.3, The Jamovi project (2024), Sydney, Australia). The Shapiro–Wilk test was used to evaluate the distribution of the continuous variables. Categorical variables are summarized by absolute and relative frequencies. Chi-squared tests were employed to compare qualitative, independent variables, such as the treatment outcome using different active units and attachments, and, if not suitable, Fisher’s exact tests were used instead. Mann–Whitney U tests were implemented to compare quantitative independent variables, such as canine alignment duration when implementing dissimilar active units and bonded attachments. The significance level was set at 0.05, two-tailed (*p* < 0.05).

## 3. Results

Treatment records of patients who consecutively visited the Postgraduate Clinic of the Department of Orthodontics of the School of Dentistry of the Aristotle University of Thessaloniki, Greece, were retrieved for the purposes of the present study. In total, upon eliminating unsuitable patients based on the abovementioned inclusion–exclusion criteria, 29 patients with 36 palatally impacted canines in the maxilla were included. The patients, at the time of the surgical procedure, had a median age of 16 years (IQR: 15–20) and the majority of them were females (n = 20, 69%) over males (n = 9, 31%). The treatment plan for all of the included patients consisted of orthodontic alignment upon surgical exposure and active traction of the impacted canine. Overall, 34 displaced canines were successfully moved orthodontically and fully aligned at the end of treatment, documenting a success rate of 94.4%, while the treatment of two canines (5.6%) was considered to have failed. In terms of duration for the successfully treated canines, the median canine alignment lasted 17.5 months (IQR: 10.3–27.8).

### 3.1. Treatment Outcome and Active Unit

The type of active unit was used to categorize the displaced canines included in the present study. The first group included the canines to which a ballista spring was employed in order to initiate active traction, while the second group comprised those whose active unit was a cantilever configuration. Patients in both groups demonstrated similar baseline characteristics in terms of age (median_BALLISTA_ = 16 years vs. median_CANTILEVER_ = 17 years) and depth of impaction. Based on Fisher’s exact test, no statistically significant difference could be detected between the two systems, pertaining to a successful treatment outcome (*p* > 0.99). The results are presented in detail in Table 1.

### 3.2. Treatment Outcome and Bonded Attachment

The treated canines were divided into two groups based on the type of attachment bonded at their palatal surface during surgical exposure, with matching ages (median_EYELET_ = 16 years vs. median_BRACKET_ = 17 years) and depths of impaction. In the first group, the attachment was a bracket, while in the second was an eyelet. According to Fisher’s exact test, successful orthodontic movement and alignment of the ectopic canines were found not to be statistically associated with the type of bonded attachment (*p* = 0.52) (Table 1).

### 3.3. Treatment Duration and Active Unit

The mechanisms implemented for applying active traction to displaced canines were compared in terms of the total time period required for canine eruption and complete alignment. Based on the results of the Mann–Whitney U test, the canine alignment duration did not significantly differentiate between the use of cantilever configuration and the ballista spring (*p* = 0.56). The results are demonstrated elaborately in Table 2.

### 3.4. Treatment Duration and Bonded Attachment

The impacted canines were divided into two categories: those whose attachment bonded at the palatal surface of the impacted canine was a bracket and those with an eyelet placed instead. As indicated by the Mann–Whitney U test, there was a statistically significant difference regarding the canine alignment duration and the type of the bonded attachment, and more specifically, brackets were found to significantly increase the time required when compared to eyelets (*p =* 0.009) (Table 2).

## 4. Discussion

This retrospective study investigated the use of two types of active units and bonded attachments as factors that potentially affect the treatment outcome and duration of the alignment of palatally impacted canines. Our findings suggest that the choice of one active unit does not significantly outmatch the other, neither in terms of the success or failure of treatment, nor pertaining to the time required for canine extrusion and alignment. Therefore, the current investigation indicates that both types of active units exhibit similar clinical performance; hence, choosing one over the other would not affect the clinician’s preference.

To the authors’ best knowledge, our investigation is the first clinical study available in the literature comparing these two specific biomechanical systems; therefore, no other data are available to support or reject our findings. The only published clinical study available referring to the use of different biomechanical systems is a clinical trial comparing K9 springs and ballista springs, documenting no significant differences regarding the successful treatment outcome and duration between the two systems [26]. Other non-clinical studies of similar context but not concerning the comparison between the active units of the present study are at one’s disposal, including a finite element analysis by Jena et al. assessing the displacement and stress distribution of different units, such as Kilroy springs, ballista springs, and temporary anchorage devices, in the course of palatally impacted canines’ traction [27] and a dentoform model-based study comparing three different force systems, consisting of Kilroy springs, elastic chains, and steel ligature wires [28]. The findings of the finite element analysis favor the use of miniscrew-assisted cantilever configurations, reporting that their implementation could provide an efficient solution for the orthodontic/surgical treatment of palatally displaced canines [27]. Based on the findings of Yadav et al.’s study, using Kilroy springs guarantees the provision of more consistent force direction, thus keeping at a minimum undesirable and potentially harmful jiggling when compared to the other force systems [28].

Based on the results of the current study, the type of attachment does not have a significant impact on treatment success; however, a finding of clinical significance seems to be the superiority of eyelets, as bonded attachments, when compared to edgewise brackets, in terms of time needed for canine extrusion and alignment. One possible explanation for the prolonged canine alignment duration could be increased soft-tissue resistance due to the higher profile of brackets as opposed to eyelets. As previously mentioned in a study conducted by Becker et al. concerning the factors associated with the successful bonding of attachments on the palatal surface of an impacted tooth, bonding eyelets had a higher success rate over conventional brackets (a 94.4% success rate over 74%). Moreover, a higher failure (debonding) rate of brackets as attachments was noted in the palatal surface in comparison to any other surface [29]. Therefore, Becker et al. indicated the superiority of eyelets as bonded attachments in the palatal surface of displaced teeth in terms of longevity of bonding [29].

### Strengths and Limitations

To the authors’ best knowledge, the findings of the current investigation are the first available pertaining to the comparison of two specific biomechanical systems for the orthodontic treatment of palatally impacted canines. More specifically, it is the first clinical study that retrospectively examines patients’ files in consecutive order, comparing cantilever configurations with ballista springs and brackets with eyelets in terms of treatment outcome and duration.

Notwithstanding the above, some limitations should be mentioned for the present study. The main limitation emanates from the study design, which is a retrospective observational design. It is common knowledge that the most reliable conclusions pertaining to clinical considerations may be drawn by conducting randomized control trials, although in some cases, observational study designs are preferred [30]. However, due to the fact that canine impaction demonstrates relatively low frequency, designing and conducting a randomized controlled clinical trial pertaining to the influence of different biomechanical systems on the treatment outcome and duration of palatally impacted canines, though constructive, would be challenging. Another limitation could be the relatively small sample size; however, as mentioned above, the current study is based on the retrospective examination of patient records from a single postgraduate clinic. Undoubtedly, further multi-center research would be necessary to withdraw more reliable and evidence-based conclusions regarding the clinical use of different biomechanical systems for the efficient management of impacted canines.

## 5. Conclusions

Based on the findings of the present study, the following can be concluded:

- Ballista springs, when applied for the active traction of palatally impacted canines, are not significantly superior over cantilever configurations in terms of both canine treatment success and alignment duration.
- The choice of the bonded attachment, though not significantly affecting the treatment outcome, may lead to an increase in the time required for the displaced canine’s alignment. More specifically, the use of brackets over eyelets significantly prolongs the canine alignment duration.

## Figures and Tables

**Table 1:** Treatment outcome with different active units and bonded attachments.

Characteristic	Failure (n = 2, 5.6%)	Success (n = 34, 94%)	Total (n = 36, 100%)	*p* Value
**Active unit**				>0.99 ^1^
Ballista	1.0 (50.0%)	22.0 (64.7%)	23.0 (63.9%)	
Cantilever	1.0 (50.0%)	12.0 (35.3%)	13.0 (36.1%)	
**Attachment**				
Bracket	1 (50%)	10 (29%)	11.0 (30.6%)	0.52 ^1^
Eyelet	1 (50%)	24 (71%)	25.0 (69.4%)	

^1^ Fisher’s exact test.

**Table 2:** Canine alignment duration with different active units and bonded attachments.

Characteristic	Alignment Duration * (n = 34)	*p* Value
**Active unit**		0.56 ^2^
Ballista (n = 22, 64.7%)	18 (10–26.8) ^1^	
Cantilever (n = 12, 35.3%)	17.5 (14–27.5) ^1^	
**Attachment**		0.009 ^2^
Bracket (n = 10, 29.4%)	32.5 (18.5–47.8) ^1^	
Eyelet (n = 24, 70.6%)	16.5 (10–21.3) ^1^	

^1^ Median (IQR). ^2^ Mann–Whitney U test. * In months.

## Data Availability

The datasets generated during and/or analyzed during the current study are available from the corresponding author upon reasonable request.
